# Understanding the Influence and Impact of Stakeholder Engagement in Patient-centered Outcomes Research: a Qualitative Study

**DOI:** 10.1007/s11606-021-07104-w

**Published:** 2022-03-29

**Authors:** Maureen Maurer, Rikki Mangrum, Tandrea Hilliard-Boone, Andrew Amolegbe, Kristin L. Carman, Laura Forsythe, Rachel Mosbacher, Julie Kennedy Lesch, Krista Woodward

**Affiliations:** 1 American Institutes for Research (AIR), Chapel Hill, NC USA; 2 Vector Psychometric Group, LLC, Chapel Hill, NC USA; 3 MBA candidate, Duke University, Fuqua School of Business, NC Durham, USA; 4 Patient-Centered Outcomes Research Institute (PCORI), 1828 L Street NW, Suite 900, Washington, DC 20036 USA

**Keywords:** patient and stakeholder engagement, comparative effectiveness research, patient-centered outcomes research, patient and public involvement

## Abstract

**Background:**

Engaging patients and other stakeholders as partners in research offers promise in improving the relevance and usefulness of research findings.

**Objective:**

To explore the influence and impact of patient and other stakeholder engagement on the planning and conduct of comparative effectiveness research studies.

**Design:**

Qualitative study with virtual, hour-long semi-structured interviews.

**Participants:**

Fifty-eight researchers and fifty-one partners from a diverse purposeful sample of fifty-eight studies funded by the Patient-Centered Outcomes Research Institute (PCORI).

**Approach:**

Content and thematic analysis of interview data.

**Key Results:**

Described as an integral, long-term part of the research process, engagement influenced all aspects of the design and execution of studies. Partner influence was also dynamic and iterative, taking different forms over the course of the study. Across studies, we identified 387 discrete examples of influence and classified each as one of five types of influence, derived inductively from the interview data: co-producing, redirecting, refining, confirming, and limited. Most projects exhibited multiple types of influence, with 50 researchers and 41 partners reporting two or more types of influence within a project. Of the 387 examples of stakeholder influence, 306 had at least one reported impact on the study. Such impacts included changes to reflect the needs and preferences of patients or clinicians, as well as impacts on study feasibility, study quality, engagement scope or quality, and study relevance. Both researchers and partners identified multiple types of impact within projects, with 42 researchers and 38 partners reporting two or more types within a project. Because of these observable impacts, researchers and partners described engagement as worthwhile.

**Conclusions:**

Findings provide insights for funders and institutions supporting engagement, measurement efforts, and clinical researchers aiming to conduct engaged research and observe similar influences and impacts in their own studies.

## INTRODUCTION

A growing body of evidence is resolving early questions concerning the feasibility and value of engaging patients and other stakeholders in the clinical research process. Engaging stakeholders promotes inclusion and partnership with individuals who bring unique perspectives and hold a direct interest in research findings. Early studies demonstrate that stakeholders engaged as research partners have influenced study protocols and study enrollment rates.^1–5^ These contributions by stakeholder partners have resulted in impacts to studies’ acceptability, feasibility, rigor, and relevance.^6^

Much of the initial discovery about how patients and other stakeholder partners impact research has been based on open-ended survey responses, study reports, or publications that included varying, researcher-generated, or nominal information about engagement. More detailed descriptions of how stakeholders influence the research process, the specific ways in which that influence affects the planning and conduct of a study, and the contributing factors and approaches to engagement that determine the nature of influence that stakeholders might have would strengthen this knowledge base. Such information can bring a deeper understanding of the specific benefits of patient and other stakeholder engagement in health research and can offer greater specificity to clinical researchers wishing to engage stakeholders.

To advance understanding of how engagement influences the research process, we report findings from in-depth interviews with researchers and partners. Research questions were as follows: (1) How did engagement influence the planning and conduct of studies? (2) What impacts on the study resulted from that influence? We defined influence as the contributions of partners in terms of behaviors, decisions, or events within individual studies. We defined impact as the results of those contributions on the study design and conduct. We conducted interviews with individuals from a diverse sample of studies from the Patient-Centered Outcomes Research Institute (PCORI) research portfolio. PCORI requires awardees to engage diverse stakeholders in comparative effectiveness research studies. Awardees have used a wide array of engagement practices, implemented with varied intensity ranging from input to consultation to collaboration to shared leadership.^4,6,7^ Consequently, this shared context in PCORI-funded studies provides a rich opportunity to describe the influence and impact of engagement on research.

## METHODS

We designed a systematic study using qualitative methods because of the need for in-depth information on influence and impact of engagement. To ensure that this work was fully informed by stakeholders, PCORI’s Advisory Panel on Patient Engagement provided input throughout all stages of the study. The American Institutes for Research’s Institutional Review Board (IRB) reviewed the protocol.

### Sample

We used purposeful sampling to achieve heterogeneous representation of PCORI’s portfolio in terms of study completion status (i.e., complete and active), funding announcement category, PCORI priority content area, study design, study populations, and health conditions.^8,9^ Active studies were eligible if they had completed at least one contract year. To optimize the chance that interview participants could speak to engagement’s influence, we prioritized studies whose principal investigators (PIs) reported in the PCORI-collected survey and administrative data that partner influence had occurred or that they had engaged different types of stakeholders across multiple study phases. From 301 eligible studies, we identified 80 for inclusion and emailed PIs requesting participation. We asked PIs to share contact information for study partners, whom we contacted separately to request participation.

The final sample comprised 58 studies. We conducted 109 interviews with 58 researchers (PIs and/or their designee from the research team) and 51 partners. Table 1 shows study and participant characteristics. Seven studies did not include a partner interview because of partner death, time since study conclusion, language preference, or lack of response to the request for contact information. Partner interviews represented a diversity of perspectives, with 21 (41%) identifying solely as patients or caregivers.

**Table 1:** Sample description

**Characteristic**	**Number (%)**
Studies (*n*=58)	
Completion status	
Active	39 (67%)
PCORI funding announcement type	
Broad (investigator-initiated applications for patient-centered comparative clinical effectiveness research aligned with priority areas)	31 (53%)
Pragmatic (applications for pragmatic clinical trials, large simple trials, or large-scale observational studies)	11 (19%)
Targeted (one-time opportunity applications on specific, high-impact topics selected in response to input from patients and other stakeholders)	16 (28%)
PCORI priority content area	
Addressing disparities	15 (26%)
Assessment of prevention, diagnosis and treatment options	15 (26%)
Communication and dissemination research	8 (14%)
Improving healthcare systems	20 (34%)
Study design	
Experimental	46 (79%)
Observational	8 (14%)
Quasi-experimental	4 (7%)
Researcher interviews (*n*=58)	
Principal investigator alone	28 (48%)
Principal investigator designee alone*	7 (12%)
Principal investigator plus other team member	23 (40%)
Partner interview participant roles (*n*=51)	
Patient or caregiver	21 (41%)
Patient advocates or members of health or patient advocacy organizations	11 (21%)
Clinicians	4 (8%)
Subject matter experts	3 (6%)
Engagement specialists	2 (4%)
Representatives of community-based organizations	2 (4%)
Multiple roles	8 (16%)

*The principal investigator requested that the project director or co-investigator complete the interview on their behalf

### Data Collection

We developed semi-structured interview protocols for hour-long interviews conducted between June 2018 and January 2019 in English by phone or video. Interviews focused on eliciting examples of partners’ influence during the study—including roles and responsibilities, behaviors, and contributions to decision making—and how engagement affected the design or conduct of the study. Interview protocols included questions about positive impacts to the study as well as unintended, undesirable, and negative impacts. Two teams of four experienced qualitative researchers interviewed researchers and partners, respectively. All were previously familiar with engagement in comparative effectiveness research. To prepare for interviews, interviewers studied available study publications and awardee reports to PCORI and then tailored interview protocols accordingly.

### Analysis

To understand the data from different perspectives and triangulate findings, we used multiple types of analysis: coding, structured memos, and content and thematic analysis of a catalog of examples.

#### Coding and Memoing

All interviews were professionally transcribed and uploaded into NVivo 12 qualitative data analysis software. Five analysts coded the data using a codebook that initially included deductive codes based on the interview protocol and attributes of the interview participant and study (e.g., interview type, health condition). Analysts applied these deductive codes and added open codes to label examples of influence. Then, analysts wrote a structured memo for each study summarizing content related to the research questions and identified quotes from both researcher and partner interviews reflecting concrete influences and impacts of engagement.

#### Cataloging Examples of Influence and Associated Impacts

We used content analysis techniques to identify concrete, discrete examples of influence in coded data and catalog them.^10^ For each example, we developed a descriptive label reflecting the main idea (e.g., partners proposed an outcome measure, partners led recruitment activity), selected illustrative quotes, identified the study phase when it occurred, and what impact, if any, resulted. We then assigned each example to one of five types of influence stakeholders exerted, which were developed inductively from a preliminary analysis of the first 24 completed summary memos and refined using the remaining transcripts. After cataloging all examples, two analysts independently reviewed the set of examples to confirm or challenge the influence type assignments. When analysts did not agree, they met to refine definitions and reach agreement on the best fit.

We also assigned each example to one or more types of impact on the study as applicable, starting with four previously identified types of impacts: study acceptability, feasibility, rigor, and relevance.^6^ We used a similar process of assigning, reviewing, and coming to agreement on examples while also inductively revising the types of impact to better reflect the interview data.

#### Analysis and Synthesis

Analysts met weekly to discuss observations, identify thematic and content patterns in the interviews, and iteratively synthesize findings across code output, summary memos, and the catalog of examples to identify patterns of overlap and divergence, explore relationships among concepts, and identify cross-cutting themes.

## RESULTS

For each research question, we identified three cross-cutting themes, which are described in the text with additional examples and illustrative quotes included in tables. Unless otherwise noted, themes were consistent across interview participant type (i.e., researchers, partners) and study characteristics (e.g., study design, PCORI content area, health condition focus).

### How Did Engagement Influence the Planning and Conduct of PCORI-Funded Studies?

Across the 58 studies, we cataloged 387 discrete examples of influence—250 from researcher interviews and 137 from partner interviews. Researchers from all studies reported at least one example. Two partners could recall engagement activities generally but could not recall specific examples of influence. Across the examples, interview participants described involvement by a variety of partners, including patients, caregivers, representatives from professional and patient and consumer advocacy organizations, clinicians, payers, healthcare administrators, and other staff at healthcare settings such as office managers or staff who work with electronic health records. The number and types of partners involved varied, with some studies including multiple types of partners and others including one or two types.

#### Described as an Integral, Long-term Part of the Research Process, Engagement Influenced All Aspects of the Design and Execution of Studies

Interview participants talked about engagement as part of the overall process that shaped the study design and conduct. The examples of influence spanned study phases and activities from designing the study to disseminating study findings. Table 2 highlights examples for each activity, such as co-writing the proposal, developing new survey items, enrolling study participants, and contributing to manuscripts. Partners influenced how researchers conceptualized the study, executed study tasks, and communicated about the study to both study participants and external audiences. Partners also addressed study-related challenges, such as issues with recruitment or low retention. In addition, different partners could be involved at different times during the study. For example, clinicians could be involved during data collection, while patients were involved during study design discussions or dissemination.

#### Partner Influence Was Dynamic, Non-linear, and Iterative, Taking Different Forms over the Course of the Study

We identified five types of influence that described how partners exerted influence, listed in order of prevalence:

- Redirecting: Partners shift the study’s direction or suggesting new plans or materials, such as different outcome measures or expanded sampling parameters.
- Co-producing: Partners and researchers work together or collaborate, including co-conceptualizing the study design, co-executing study tasks, or having partners lead tasks.
- Refining: Partners edit or modify existing plans or materials, such as recruitment materials or manuscripts.
- Limited or no influence: Influence was constrained or did not occur because researchers were not able to implement partner suggestions (e.g., due to institutional review board constraints); researchers encountered challenges balancing stakeholder influence with science best practices; researchers did not ask for or apply partner input; or partners felt they did not have much input to offer.
- Confirming: Partners validate existing plans or materials.

Table 3 provides examples of each type of influence and the frequency of reports by interview participants. Table 5 highlights an illustrative quote for each type.

**Table 2:** Examples of partner influence showing how engagement was integral to all study activities

**Study activity**	**Examples of partner influence**
Research questions and plans	• Co-developed the study in initial project phases • Collaborated with researchers to determine priorities and suitable research questions • Co-wrote proposal for project funding • Confirmed that the study was important, feasible, timely, or worthwhile
Study design	• Discussed iteratively potential designs and endpoints • Adapted study design to address challenges and obstacles prior or during implementation • Identified weaknesses in the proposed design and helped determine how to address them • Challenged the use of a randomized controlled trial study design because partners wanted all patients to receive the benefits of the intervention • Helped determine inclusion/exclusion criteria by suggesting expansion of age range, diagnostic criteria, or additional groups (e.g., rural) • Agreed with and validated aspects of the study design
Outcome and measurement approaches	• Disputed research team’s choices and proposed alternatives for study outcomes • Helped to prioritize among desirable outcomes to study • Identified gaps in available instruments and new or additional domains for inclusion • Developed new surveys or survey items • Advised on the content or phrasing of surveys or survey items • Tested candidate instruments • Evaluated the feasibility, usability, and burden of selected measures
Intervention design and implementation	• Influenced the content and format of intervention materials (e.g., videos, apps, websites) by adding or removing content, making language patient-friendly, or making materials easier to use in the field or doctor’s office • Adapted interventions to fit study sites, clinic workflow, or patient experience • Trained intervention providers • Led intervention activities
Recruitment and enrollment processes and materials	• Conducted outreach activities to sites and patients • Created flyers, letters, and informed consent materials • Advised and cautioned researchers how content, messaging, or language might be misinterpreted during recruitment • Edited materials to make them shorter and clearer and improve cultural appropriateness • Enrolled patients in the study
Retention approaches	• Conducted retention activities, such as follow-up phone calls • Developed strategies for encouraging retention • Suggested adding incentives to prevent attrition • Created or directed the creation of tools to engage study participants, such as a newsletter or a Facebook group
Data collection	• Conducted interviews • Provided technical assistance to study sites • Facilitated communication between sites and researchers
Data analysis	• Supplied context to explain study results • Offered alternate interpretations of study results • Suggested conducting additional analyses
Dissemination	• Brainstormed and planned for dissemination, including offering advice on vehicles • Created dissemination products, such as presentations or manuscripts • Participated in dissemination activities
Engagement	• Designed or led engagement • Developed or requested alternative engagement strategies • Created written guidance and tools to support engagement • Suggested and recruited new partners

**Table 3:** The dynamic influence of engagement on PCORI-funded research: definitions, examples, and frequency of types of influence by researcher and partner interview participants

**Types**	**Examples**	**Frequency of report**		
		**Researcher,** ***n*=58**	**Partner,** ***n*=51**	**Total,** ***n*=109**
Redirecting: Partners shift the study’s direction or create new plans or materials	• Disputed research team’s choices and proposed alternatives for outcomes or measures • Recommended different recruitment strategies • Suggested new avenues for dissemination	39 (67%)	42 (82%)	81 (74%)
Co-producing: Partners and researchers work together or collaborate	• Approached researchers with study idea • Developed the intervention for the study • Led study recruitment	42 (72%)	33 (65%)	75 (69%)
Refining: Partners edit or modify existing plans or material	• Edited study materials or manuscripts • Suggested improvements to study implementation procedures	47 (81%)	24 (47%)	71 (65%)
Limited or no influence: Researchers were unable to implement partner suggestions	• IRB constraints limited ability to implement suggestions • Researchers decided not to implement • Partners felt they did not have much to contribute	20 (34%)	16 (31%)	36 (33%)
Confirming: Partners validate existing plans or materials	• Validated that study aims were important • Reviewed study recruitment materials	8 (14%)	4 (8%)	12 (11%)

Note: Types of influence listed in order of frequency of report (i.e., total number of researchers and partners who reported it)

The number of types of influences per study ranged from one to five, with 50 researchers and 41 partners reporting two or more types of influence within a single study. Sometimes, these influences were iterative, driving a cycle of influence where redirecting could lead to later refining or confirming. For example, in one study, early redirection by stakeholders resulted in a well-planned data collection protocol that required minimal refinement prior to implementation.

#### Partners Influenced Studies by Teaching Researchers About Patients’ Lives or How Other Stakeholders Carry Out Their Work

For example, partners taught researchers about what life is like for people with depression or what clinic workflow is like for a doctor. One researcher said:

[Partners are] a component to understanding the experience of the patient, how doctors perceive what we’re trying to do, how best to reach them, how best to get in touch with them…how we’re actually going to roll this out at the end of the project. It’s something—you can’t really do in a vacuum.

Another said, “We originally anticipated approaching and trying to recruit patients in-person...But what we learned in talking with this clinic was that many of their patients are farmers that live two and three hours away.”

This teaching could result in any type of influence from redirecting to confirming. Researchers responded by making changes, such as to data collection procedures, even when partners were not directly involved in that part of the study. Researchers even mentioned longer-term changes, such as changing their approach to research and altering priorities for future studies.

### What Impacts on the Study Resulted from that Influence?

Of the 387 influence examples, 306 had at least one identifiable impact on the study—208 from researcher interviews and 98 from partner interviews.

#### Researchers and Partners Reported that Engaging Patients and Other Stakeholders Had a Multi-faceted Impact on Study Planning and Conduct

In one example, a researcher summarized the multiple impacts as follows: “The study got done. We exceeded expectations…We recruited more than we expected, we enrolled more than we expected. It was phenomenal from that frame.”

From the examples, we identified five types of impact, listed in order of prevalence:

- User-centeredness and acceptability: How well the study design and study materials reflected the needs and preferences of patients, providers, or other partners
- Study feasibility: How well the team was able to execute study activities in a timely, cost-effective way
- Study quality: Changes to quality of study design (e.g., comprehensiveness, rigor) and study materials
- Engagement scope and quality: How well the study engaged diverse stakeholders across study activities
- Relevance: Usefulness of study results to intended audiences

Table 4 provides examples for each type and frequency of report by interview participants. Table 5 highlights an illustrative quote for each type of impact.

**Table 4:** How engagement shapes PCORI-funded studies: definitions, examples, and frequency of types of impact reported by researcher and partner interview participants

Types	Examples	Frequency of report		
		Researcher, *n*=58	Partner, *n*=51	Total, *n*=109
User-centeredness and acceptability: How well the study and study materials reflected the needs and preferences of patients, providers, or other partners	• Prioritized outcomes that matter to patients • Reduced burden for study site staff • Reflected user preferences for study implementation • Address lack of interest from patient populations or sites	54 (93%)	44(86%)	98 (90%)
Study feasibility: How well the team was able to execute study activities in a timely, cost-effective way	• Improved ability to collect data • Improved recruitment process and materials • Changes to project management, such as increasing or decreasing costs	41 (70%)	32 (55%)	73 (67%)
Study quality: Changes to quality of study design and study materials	• Improved study’s comprehensiveness • Changes to rigor of study design • Improved quality of intervention materials	43 (74%)	27 (53%)	70 (64%)
Engagement scope and quality: How well the study engaged diverse stakeholders across study activities	• Improved representation by expanding the number or types of stakeholders involved • Improved engagement processes	22 (38%)	11 (21%)	33 (30%)
Relevance: Usefulness of study results to intended audiences	• Validated that study aims and outcomes were important and meaningful • Increased receptivity of study findings	10 (17%)	2 (4%)	12 (11%)

Note: Types of impact listed in order of frequency of report (i.e., total number of researchers and partners who reported it)

**Table 5:** Illustrative quotes for types of engagement influence and impact

**Type**	**Illustrative quote**
**Influence**	
**Redirecting**	“People had problems with [blood samples being sent away for analysis]. They didn’t want to have their blood go out from their community. Based on the suggestion from …the advisory panel, we decided that we will work with the local health services where they will do the lab work.” (Researcher)
**Co-producing**	“From the very first glimmer, I was involved. We sketched out what a trial would look like together and wrote the grant together.” (Partner)
**Refining**	“We were missing eligible patients because of a [four-week screening] window. All these clinicians realized that and said, ‘let’s change the protocol.’ And we did make that window bigger.” (Researcher)
**Limited or no influence**	“We had an online platform that we wish we would have had a little more involvement with the development, because it was an issue with a lot of our patients using that platform.” (Partner)
**Confirming**	“It was a complicated study. When we initially came up with the idea, we weren’t sure that it was even going to be feasible. But after again engaging the stakeholders…we were reassured and encouraged to move forward with it.” (Researcher)
**Impact**	
**User-centeredness and acceptability**	“Another challenge [the researchers had was] making this questionnaire user-friendly, not asking too invasive questions … we gave them suggestions from a patient standpoint.” (Partner)
**Study feasibility**	“The survey questionnaires were coming in at a slower pace than what the researchers wanted… [After we gave our suggestions], they realized a huge difference. They mentioned something like 80, 90 percent [up] from 50 percent.” (Partner)
**Study quality**	“The stakeholders gave us insights and suggestions that we probably wouldn’t have come up with on our own that in the end improved the trial design, trial implementation, and, hopefully, will give us the most meaningful results.” (Researcher)
**Engagement scope and quality**	“We worked together and created a patient partner guide and a handbook and a glossary … It’s something that we will have to continue to refine and change to make it as user friendly as possible.” (Partner)
**Relevance**	“We published an article that documented the results of our focus groups and key informant interviews with patients and caregivers, and through their [partners] assistance, we ended up getting an Altmetric score that placed us in the top 5% of all medical articles published….I attribute it to what they did.” (Researcher)

The number of reported impacts per project ranged from one to five, with 42 researchers and 38 partners specifying two or more types of impact within a project. Most reported impacts affected the study in ways that researchers and partners wanted. For example, partner input improved enrollment or the study’s comprehensiveness. However, a few examples resulted in undesirable impacts such as increased costs or delays. In these instances, researchers described needing additional time or resources to incorporate partner suggestions. Partners shared examples of when researchers failed to listen to or invite partner suggestions early, which resulted in the need for extensive revisions later that used up time and resources.

#### Not All Partner Influence Resulted in an Observable Impact on the Study

About 20% of the examples of influence did not have a reported, observable impact. These included examples where participants did not yet know the impact since the study was not complete, or partners did not know how their contributions had affected the study. Also, when influence was limited, it often resulted in no impact.

#### Because of Observable Impacts, Researchers and Partners Described Engagement as Worthwhile

Researchers stated that, although engagement took time and effort, it yielded a valuable impact on the study. For example, one researcher said: “At first we viewed it as burdensome, but over time we really started to see the value in the way it was impacting the decisions we were making and how we were carrying out the study so that it would be more relevant to patients and providers on the front lines.” As a result, researchers said they were more likely to seek stakeholder input on study proposals and implementation problems in the future. Partners also valued the opportunity to have an impact on studies. For example, one partner said, “This study has opened my eyes personally to how important my input is. I did not know that until I got involved with this study, how important a patient’s voice is in studies.”

## DISCUSSION

These findings add a new level of understanding about both engagement and the dynamic nature of partnership in patient-centered research. Findings confirm earlier analyses of the benefits of patient and other stakeholder engagement in research and that active influence of partners most often improved studies’ feasibility and acceptability.^2,6^ Findings also offer new insights about how influence happens, show the diversity of influences that stakeholders have, and suggest additional impacts than what has been previously documented, including on engagement approaches.

The ways in which partners exerted influence on studies—by redirecting, co-producing, refining, or confirming—were dynamic, non-linear, and iterative, driving a cycle of influence across the stages of a study. Although these types of influence show conceptual similarity to the continuum of engagement identified in other frameworks, the underlying concepts differ.^11^ Because each study reported multiple types of influence, classifying an individual study to one type of influence—or a single place on the continuum of engagement—was not possible. Study teams could establish a “co-producing relationship” that could still have refining, redirecting, or confirming instances of influence. Similarly, study teams that initially engaged with partners as consultants could be transformed by early interactions, eventually amassing multiple examples of co-producing influence. In this way, engagement evolves throughout the lifecycle of study—weaving throughout the continuum of engagement in response to study needs. The discovery of iterative influence suggests that initial opportunities for stakeholder influence may generate future, though different, forms of influence, all of which increases the likelihood of desirable impacts on the study’s overall success.

It is worth noting that not all instances of influence resulted in a desirable, observable, or known impact on the study. The few reported undesirable impacts related to unanticipated delays or increased costs. Sometimes, researchers unintentionally constrained influence by not offering an opportunity or clearly articulating a role for stakeholders. In other cases, researchers could not implement stakeholders’ suggestions due to IRB constraints or other barriers. This finding adds weight to early conceptualizations of engagement that suggest that human and system factors can enable or disable partner contributions.^11–13^ Institutional system adjustments and training for researchers and partners in engagement may be warranted. For example, researchers and partners may need additional support balancing input and scientific rigor in team discussions.

Many impacts identified by this study were proximal in nature. This focus is consistent with the fact that two-thirds of the studies in the sample were in progress and proximal impacts may be easiest to recognize and recall. It will be important, as a greater number of stakeholder-driven studies are completed and diffused, to also measure longer-term impacts, such as the relevance and usefulness of findings in healthcare decision making. Doing so requires having a comprehensive way of capturing proximal impacts as they are occurring. The taxonomy of types of influences and impacts from this study can be foundational to future research in this area.

### Limitations and Strengths

We used a purposeful sampling approach with PCORI-funded studies and conducted interviews in English, which may limit generalizability of the findings. For each study, we interviewed one partner and one to two researchers; other people involved in the study may have had different experiences. However, across studies, we were able to identify diverse perspectives, including patients, clinicians, and other stakeholders. Because interviewers were outside of the funder, participants had greater assurance of confidentiality to share less socially biased examples.

At times, interview participants had difficulty recalling specific examples of influence and impact. Because the analysis rested on recalled examples, it may not capture the full breadth of influences or impacts. To enhance recall, interviewers prompted participants to think back to specific events or decision-making points mentioned in study progress reports and asked them to share what they remembered. Even with the limitations, these systematic, in-depth qualitative findings lay a critical foundation for continued examination of engagement’s influence and impact.

### Implications

For funders and institutions, these findings continue to strengthen the evidence for investing in and supporting engagement in research, including further research on how to engage effectively to achieve and amplify these types of influences and impacts. The evidence for engagement now stands at a critical juncture—moving from describing the feasibility of engagement and the possible ways engagement can influence and impact studies to recommending how stakeholders *should be* engaged. To do so, direction for future research necessitates understanding what engagement approaches work best for whom at different stages of the research process, as well as the longer-term impacts of engagement on the uptake of research findings and ultimately health and health care.

These findings also have implications for measuring engagement processes and outcomes. There is a lack of useful, validated measures. Early measures may be overly simplistic by classifying levels of engagement to entire studies or perceiving engagement as linear. This study suggests that measuring engagement is more complex and will need to capture the multiple types of influence occurring within individual studies and how these different types iteratively build on each other.

Finally, for clinical researchers wishing to realize influences and impacts in their own studies, these findings also offer insights. For engagement to be successful, previous research has highlighted the importance of clarifying roles and expectations for stakeholder partners and ensuring that partners understand the value of their efforts to the project.^14^ These findings offer additional insights to assess the progress and success of engagement, including whether engagement is yielding the desired influences and impacts on the study. For example, if partners are only confirming or refining existing plans or materials, studies may not be realizing the full potential of engagement. Researchers may need to build in opportunities for, pay attention to, and respond to stakeholders’ redirection; extend an invitation to co-execute study activities; or create opportunities for partners to lead and innovate on study tasks. Taking time to assess stakeholders’ interest in influencing the study and shaping opportunities accordingly engenders trust and authentic partnership.

Taken as a whole, the findings suggest that the actions of funders and institutions to support engagement, efforts to improve measurement, and the formation of active partnerships between researchers and stakeholders are necessary to see the impacts we observed in this study in health research more broadly and ultimately move towards a culture of engagement.
